# Differences and discrepancies between 2005 and 2008 Abbreviated Injury Scale versions - time to standardise

**DOI:** 10.1186/1757-7241-20-11

**Published:** 2012-02-02

**Authors:** Kjetil G Ringdal, Morten Hestnes, Cameron S Palmer

**Affiliations:** 1 Department of Research, Norwegian Air Ambulance Foundation, P.O. Box 94, N-1441 Drøbak, Norway; 2 Division of Emergencies and Critical Care, Oslo University Hospital - Ullevål, Kirkeveien 166, N-0450 Oslo, Norway; 3 Institute of Clinical Medicine, Faculty of Medicine, University of Oslo, Kirkeveien 166, N-0450 Oslo, Norway; 4 Oslo University Hospital Trauma Registry, Department of Research and Development, Division of Emergencies and Critical Care, Oslo University Hospital - Ullevål, Kirkeveien 166, N-0450 Oslo, Norway; 5 Trauma Service, The Royal Children's Hospital Melbourne, Flemington Rd, Parkville 3052, Australia; 6 Department of Epidemiology & Preventive Medicine, Monash University, 99 Commercial Road, Melbourne 3004, Australia

## Abstract

The aim of this letter is to facilitate the standardisation of Abbreviated Injury Scale (AIS) codesets used to code injuries in trauma registries. We have compiled a definitive list of the changes which have been implemented between the AIS 2005 and Update 2008 versions. While the AIS 2008 codeset appears to have remained consistent since its release, we have identified discrepancies between the codesets in copies of AIS 2005 dictionaries. As a result, we recommend that use of the AIS 2005 should be discontinued in favour of the Update 2008 version.

## Correspondence

In order for trauma registry data to be comparable across institutions and trauma systems, the injury classification systems which underpin them must be comparable and consistent. In most trauma registries, injuries are classified using the Abbreviated Injury Scale (AIS) [1,2]. AIS-derived scores such as the Injury Severity Score (ISS) [3] and New Injury Severity Score [4] are used to quantify the severity of (and compare) multiply injured patients; to select patients for inclusion in registries; and as part of the definitions used to describe major trauma. Consequently, consistency of the AIS codesets used is pivotal to the purpose and validity of trauma registries.

The Association for the Advancement of Automotive Medicine (AAAM) has updated and maintained the AIS since the early 1970s. Since its initial publication, the AIS codeset has expanded and evolved over several editions. The current version of the AIS (AIS08) [2] is a 2008 update of the greatly expanded 2005 edition (AIS05) [1]. The changes implemented between AIS05 and AIS08 are known to be comparatively minor [5,6]. However, the effect of these changes on actual datasets has not been assessed.

Between the 2005 and 2008 AIS releases, the AAAM released an unknown number of periodic updates. These contained individual AIS dictionary pages on which one or more AIS codes had been updated, with the intent that they could replace earlier versions of the pages in users' AIS05 dictionaries. However, it was not always clear which codes were updated on each page, despite this being crucial for users of electronic versions of the AIS. Also, if users purchased AIS dictionaries during this gradual update process or did not update their dictionaries over time, it is possible that not all AIS05 or AIS08 dictionaries in use contain the same codesets or coding instructions.

Consequently, we aimed to identify all of the changes made to the AIS codeset since 2005. We therefore evaluated all of the codeset updates (additions, modifications and deletions), as well as any instruction changes made between the 'original' AIS05 and the final 'updated' AIS08. In aiming to develop a list specifying these updates, we also assessed whether any codeset inconsistency exists between copies of the AIS dictionary.

## Evaluation

Between the three authors of this letter and the available online sources, we obtained nine separate AIS documents. These documents are summarised in Table 1. We evaluated three published AIS05 dictionaries (referred to as '2005-A', '2005-B' and '2005-C'), two online periodic AIS updates ('2005-D' [7] and '2005-E' [8]), and four published AIS08 dictionaries ('2008-A' through '2008-D'). The seven published AIS05 and AIS08 dictionaries were purchased at different times between 2005 and 2011; three separate revision numbers were identified amongst these dictionaries.

**Table 1:** AIS dictionaries and AAAM updates used in evaluating AIS codeset change and consistency

AIS05 dictionaries and updates			
**Dictionary code**	**Author owning the dictionary**	**Dictionary revision number**	**Year dictionary obtained/*released***

**2005-A**	MH	None	2005
**2005-B**	CSP	None	2006
**2005-C**	KGR	01/2008	2008
***2005-D ***[7]	*-*	*(June 2006)*	*2006*
***2005-E ***[8]	*-*	*(March 2007)*	*2007*

**AIS08 dictionaries**			

**Dictionary code**	**Author owning the dictionary**	**Dictionary revision number**	**Year dictionary obtained**

**2008-A**	MH	10/2008	2008
**2008-B**	CSP	10/2008	2009
**2008-C**	KGR	10/2008	2010
**2008-D**	CSP	10/2008	2011

Three AIS05 and four AIS08 dictionaries in the possession of listed authors are described in terms of the revision number printed in the front of the dictionary, and the year in which the copy of the dictionary was obtained. Two AAAM periodic updates (italicised) are also described.

All of the available AIS data sources were carefully compared to determine which differences existed between AIS dictionaries. Firstly, each author independently compared their own AIS05 and AIS08 dictionaries; next, identified differences were discussed, and all changes identified by any author were re-assessed in each dictionary. The online data sources were also compared with differences identified between dictionaries, as well as being used to identify additional changes for checking against the published AIS dictionaries.

The complete list of codeset, instruction and mapping changes identified between AIS05 and AIS08 is available online (http://www.rch.org.au/paed_trauma/database.cfm). A total of 80 changes were made between AIS05 and AIS08, of which 31 involve changes to AIS codes or maps and 49 involve changes to wording or instructions. Further information regarding the results of our evaluation may be found in Additional File 1.

All four of the AIS08 dictionaries reviewed contained exactly the same codeset. By contrast, no two of the AIS05 data sources evaluated contained exactly the same list of AIS codes. In addition, all of the AIS05 sources contained at least some 'updated' codes (as defined by whether the code was contained in the AIS08 codeset). There was also some independence between when AIS05 dictionaries and updates were produced, and which (or how many) updated codes they contained - while the AIS05 source from early 2008 ('2005-C') included the most updated codes, the 2005 source ('2005-A') included more updates than either of the sources produced in 2006 ('2005-B' and '2005-D').

## Discussion

All of the data sources reviewed were found to contain at least some of the updated AIS codes which were introduced between 2005 and 2008. Conversely, none of the data sources contained the complete ('original') codeset of AIS05 codes (that is, the AIS codeset which does not contain any of the updates contained in AIS08). It can consequently be concluded that in practice, any registry using AIS05 is likely to have a codeset which differs slightly from other registries using AIS05. By contrast, the AIS08 appears to have been completely stable since its release in late 2008.

The differences between the AIS05 and AIS08 codesets are small in the context of the overall AIS codeset. However, the effects of these codeset changes in practice have not been formally assessed, and it is known that even minor AIS codeset change can disproportionally affect summary scores such as the ISS [9]. Also, our findings are particularly relevant to the issue of mapping data between different AIS versions. This requires absolute consistency of both the original and updated AIS codesets being employed in order to be feasible, as errors caused by codes missing from the maps disproportionately affect the time required to perform accurate and complete mapping. As a result, AIS mapping involving AIS05 is likely to be problematic.

The concept of periodic updates (to provide for the most contemporary evaluations of injury severity) is not unsound. A comparable example would be yearly updates which are produced for the clinical modifications of International Classification of Diseases (ICD) codes in some countries. The community of AIS users, though, is not always well-linked, and many users do not have any regular contact with the AAAM. As a result, ad hoc updates of the AIS do not appear to be helpful, and we would suggest that only full updates should be published.

In summary, we believe it is intuitive that injury coding should be consistent to enable trauma data to be comparable between institutions. We have shown that the AIS05 should not be regarded as a separate version for coding or comparative purposes. In the interests of codeset standardisation, we recommend that use of the AIS05 should be discontinued in favour of the consistent AIS08 codeset.

## List of abbreviations used

AAAM: Association for the Advancement of Automotive Medicine (formerly the American Association for Automotive Medicine); AIS: Abbreviated Injury Scale; AIS05: The Abbreviated Injury Scale 2005 [1]; AIS08: The Abbreviated Injury Scale 2005 - Update 2008 [2]; ICD: International Classification of Diseases; ISS: Injury Severity Score [3]

## Competing interests

The authors declare that they have no competing interests.

## Authors' contributions

This manuscript was jointly conceived by all authors. Differences between codesets were identified by all authors, and the manuscript and additional file were edited, reviewed and approved by all authors following both initial drafting by CSP, and re-formatting by KGR.

## Supplementary Material

Additional file 1 **AIS codes changing between 2005 and 2008 codesets, illustrating which codes were present in available dictionaries**. The table lists all 31 AIS codes which changed, were introduced or had map changes between AIS05 and AIS08. The versions of each code present in the nine dictionaries and data sources available to the authors are shown. Click here for file

## References

[B1] Gennarelli TA, Wodzin E The Abbreviated Injury Scale 2005 2005 Barrington, IL: Association for the Advancement of Automotive Medicine

[B2] Gennarelli TA, Wodzin E The Abbreviated Injury Scale 2005 - Update 2008 2008 Barrington, IL: Association for the Advancement of Automotive Medicine

[B3] Baker SP O'Neill B Haddon W Jr Long WB The injury severity score: a method for describing patients with multiple injuries and evaluating emergency care J Trauma 1974 14 187 196 10.1097/00005373-197403000-00001 4814394

[B4] Osler T Baker SP Long W A modification of the injury severity score that both improves accuracy and simplifies scoring J Trauma 1997 43 922 925 10.1097/00005373-199712000-00009 9420106

[B5] Palmer CS Niggemeyer LN Charman D Double coding and mapping using Abbreviated Injury Scale 1998 and 2005: Identifying issues for trauma data Injury 2010 41 948 954 10.1016/j.injury.2009.12.016 20074729

[B6] Tohira H Jacobs I Matsuoka T Ishikawa K Impact of the version of the Abbreviated Injury Scale on injury severity characterization and quality assessment of trauma care J Trauma 2011 71 56 62 10.1097/TA.0b013e31821e5a25 21818015

[B7] AIS Changes - June06 http://www.aaam1.org/ais/AISchanges_June06.pdf retrieved 16 January 2012. Association for the Advancement of Automotive Medicine, 2006

[B8] AIS Changes - March07 http://www.aaam1.org/ais/AISHighchanges_Mar07.pdf retrieved 16 January 2012. Association for the Advancement of Automotive Medicine, 2007

[B9] Skaga NO Eken T Hestnes M Jones JM Steen PA Scoring of anatomic injury after trauma: AIS 98 versus AIS 90 - do the changes affect overall severity assessment? Injury 2007 38 84 90 10.1016/j.injury.2006.04.123 16872609

