# Supplementary material for: Differences and discrepancies between 2005 and 2008 Abbreviated Injury Scale versions - time to standardise
Source: Scand J Trauma Resusc Emerg Med. 2012 Feb 2;20:11. doi: 10.1186/1757-7241-20-11 (PMC3352314; doi:10.1186/1757-7241-20-11)
Supplement: Additional file 1 — AIS codes changing between 2005 and 2008 codesets, illustrating which codes were present in available dictionaries. The table lists all 31 AIS codes which changed, were introduced or had map changes between AIS05 and AIS08. The versions of each code present in the nine dictionaries and data sources available to the authors are shown. [file 1757-7241-20-11-S1.PDF]

Differences and discrepancies between 2005 and 2008 Abbreviated Injury Scale versions - time to standardise

Additional file 1. AIS codes changing between 2005 and 2008 codesets, illustrating which codes were present in available dictionaries.

AIS codes and brief descriptors are provided both for AIS05 codes ('Original') and their AIS08 equivalents ('Updated'). For the nine available AIS05 and AIS08 data sources summarised in Table 1, the AIS code versions used ('Original' or 'Updated') are listed. Where a code did not exist in AIS05 and was not found in an available data source, it is listed as having used the AIS05 codeset ('Original'). Where an AAAM AIS05 update (codes '2005-D' and '2005-E') did not contain the page for a particular AIS code, the original code version was assumed to have been used ('[Original]'). Inclusion of AIS05 codes in each data source is highlighted in green text, and of AIS08 codes in red text.

AIS05 codes *('Original')*		Status of AIS08 code compared to AIS05 code	AIS08 codes *('Updated')*		Version of AIS code present in dictionaries and updates available to authors								
					AIS05 dictionaries and updates					AIS08 dictionaries			
AIS05 code	AIS05 descriptor		AIS08 code	AIS08 descriptor	2005-A	2005-B	2005-C	2005-D [7]	2005-E [8]	2008-A	2008-B	2008-C	2008-D
120099.3	Intracranial vascular injury	Changed level	120099.9	Vascular Injury in Head NFS	*Original*	*Original*	*Original*	*Original*	*[Original]*	*Updated*	*Updated*	*Updated*	*Updated*
230299.2	Optic Nerve NFS	Changed level	230299.1	Optic Nerve NFS	*Updated*	*Original*	*Updated*	*[Original]*	*Updated*	*Updated*	*Updated*	*Updated*	*Updated*
241006.1	Retina detachment NFS	Changed level	241006.2	Retina detachment NFS	*Updated*	*Original*	*Updated*	*[Original]*	*Updated*	*Updated*	*Updated*	*Updated*	*Updated*
241008.1	Retina detachment with macula attached	Changed level	241008.2	Retina detachment with macula attached	*Updated*	*Original*	*Updated*	*[Original]*	*Updated*	*Updated*	*Updated*	*Updated*	*Updated*
241010.1	Retina detachment with macula detached	Changed level	241010.2	Retina detachment with macula detached	*Updated*	*Original*	*Updated*	*[Original]*	*Updated*	*Updated*	*Updated*	*Updated*	*Updated*
(None)	*(None)*	New code in AIS08	243199.1	Palate NFS	*Original*	*Original*	*Original*	*[Original]*	*[Original]*	*Updated*	*Updated*	*Updated*	*Updated*
243000.1	Palate - laceration	Changed format	243101.1	Palate - laceration	*Original*	*Original*	*Original*	*[Original]*	*[Original]*	*Updated*	*Updated*	*Updated*	*Updated*
(None)	*(None)*	New code in AIS08	243102.2	Palate fracture	*Original*	*Original*	*Original*	*[Original]*	*[Original]*	*Updated*	*Updated*	*Updated*	*Updated*
250699.1	Mandible fracture NFS	Changed format	250600.1	Mandible fracture NFS	*Original*	*Original*	*Original*	*Original*	*Original*	*Updated*	*Updated*	*Updated*	*Updated*
250600.1	Mandible fracture - closed or NFS	Changed format	250602.1	Mandible fracture - closed but NFS as to site	*Original*	*Original*	*Updated*	*Original*	*Updated*	*Updated*	*Updated*	*Updated*	*Updated*
(None)	*(None)*	New code in AIS08	251205.2	Multiple fractures of same orbit, closed or NFS	*Original*	*Original*	*Original*	*Original*	*[Original]*	*Updated*	*Updated*	*Updated*	*Updated*
(None)	*(None)*	New code in AIS08	251206.2	Multiple fractures of same orbit open	*Original*	*Original*	*Original*	*Original*	*[Original]*	*Updated*	*Updated*	*Updated*	*Updated*
(None)	*(None)*	New code in AIS08	410102.2	Pectoral muscle tear; laceration	*Original*	*Original*	*Original*	*Original*	*[Original]*	*Updated*	*Updated*	*Updated*	*Updated*
(None)	*(None)*	New code in AIS08	442502.2	Thymus laceration; perforation	*Original*	*Original*	*Original*	*[Original]*	*[Original]*	*Updated*	*Updated*	*Updated*	*Updated*
(None)	*(None)*	New code in AIS08	540322.2	Appendix laceration; perforation	*Original*	*Original*	*Original*	*Original*	*[Original]*	*Updated*	*Updated*	*Updated*	*Updated*
540610.1	Bladder contusion; hematoma [OIS I]	Changed level	540610.2	Bladder contusion; hematoma [OIS I]	*Original*	*Original*	*Original*	*Updated*	*[Original]*	*Updated*	*Updated*	*Updated*	*Updated*
650208.3	Dislocation - atlanto-occipital	Changed level	650208.2	Dislocation - atlanto-occipital	*Original*	*Original*	*Updated*	*Original*	*[Original]*	*Updated*	*Updated*	*Updated*	*Updated*
(None)	*(None)*	New code in AIS08	711010.5	Amputation at shoulder - bilateral	*Original*	*Original*	*Original*	*[Original]*	*[Original]*	*Updated*	*Updated*	*Updated*	*Updated*
(None)	*(None)*	New code in AIS08	711012.5	Amputation at or above elbow, below shoulder - bilateral	*Original*	*Original*	*Original*	*[Original]*	*[Original]*	*Updated*	*Updated*	*Updated*	*Updated*
720499.1	Axillary vein NFS	Changed level	720499.2	Axillary vein NFS	*Updated*	*Original*	*Updated*	*[Original]*	*Updated*	*Updated*	*Updated*	*Updated*	*Updated*
(None)	*(None)*	New code in AIS08	740401.1	Muscle tear partial disruption	*Original*	*Original*	*Original*	*[Original]*	*Original*	*Updated*	*Updated*	*Updated*	*Updated*
(None)	*(None)*	New code in AIS08	740403.2	Muscle tear complete disruption	*Original*	*Original*	*Original*	*[Original]*	*Original*	*Updated*	*Updated*	*Updated*	*Updated*
752271.2	Radius shaft fracture - complex; comminuted; segmental	Changed map	*AIS code is the same in AIS08, but map has been changed in column '⇒AIS98' from 752800.2 to 752804.3*		*Updated*	*Original*	*Updated*	*[Original]*	*Updated*	*Updated*	*Updated*	*Updated*	*Updated*
(None)	*(None)*	New code in AIS08	811010.5	Amputation at hip or buttock - bilateral	*Original*	*Original*	*Original*	*[Original]*	*[Original]*	*Updated*	*Updated*	*Updated*	*Updated*
(None)	*(None)*	New code in AIS08	811012.5	Amputation at or above knee, below hip - bilateral	*Original*	*Original*	*Original*	*[Original]*	*[Original]*	*Updated*	*Updated*	*Updated*	*Updated*
854455.2	Fibula fracture below ankle joint - lateral and medial malleoli (bimalleolar)	Changed map	*AIS code is the same in AIS08, but map has been added in column '⇐AIS98'*		*Updated*	*Original*	*Updated*	*Original*	*Updated*	*Updated*	*Updated*	*Updated*	*Updated*
854456.2	Fibula fracture below ankle joint - lateral and medial malleoli (bimalleolar) - open	Changed level	854456.3	Fibula fracture below ankle joint - lateral and medial malleoli (bimalleolar) - open	*Original*	*Updated*	*Updated*	*Updated*	*Original*	*Updated*	*Updated*	*Updated*	*Updated*
854463.2	Fibula fracture through ankle joint - lateral and medial malleoli (bimalleolar)	Changed map	*AIS code is the same in AIS08, but map has been added in column '⇐AIS98'*		*Updated*	*Original*	*Updated*	*Original*	*Updated*	*Updated*	*Updated*	*Updated*	*Updated*
854464.2	Fibula fracture through ankle joint - lateral and medial malleoli (bimalleolar) - open	Changed level	854464.3	Fibula fracture through ankle joint - lateral and medial malleoli (bimalleolar) - open	*Original*	*Updated*	*Updated*	*Updated*	*Original*	*Updated*	*Updated*	*Updated*	*Updated*
(None)	*(None)*	New code in AIS08	854465.2	Fibula fracture - trimalleolar	*Updated*	*Original*	*Updated*	*Original*	*Updated*	*Updated*	*Updated*	*Updated*	*Updated*
(None)	*(None)*	New code in AIS08	854466.3	Fibula fracture - trimalleolar - open	*Updated*	*Original*	*Updated*	*Original*	*Updated*	*Updated*	*Updated*	*Updated*	*Updated*
